# Management of the endodontic-periodontal lesion

**DOI:** 10.1038/s41415-025-8327-x

**Published:** 2025-04-11

**Authors:** Ayman Al-Sibassi, Sadia Ambreen Niazi, Peter Clarke, Adejumoke Adeyemi

**Affiliations:** 476692068409202019464 School of Dentistry, University of Liverpool, Liverpool, United Kingdom; 571725409332855173583 Department of Endodontics, Centre of Oral, Clinical and Translational Sciences, Faculty of Dentistry, Oral and Craniofacial Sciences, King´s College London, London, United Kingdom; 386183729308414059885 Department of Restorative Dentistry, University Dental Hospital of Manchester, United Kingdom

## Abstract

Endodontic-periodontal lesions (EPLs) develop due to the various pathways that allow microbial migration between these two compartments. The authors review the historical and current research on the aetiology, diagnostic pathways, prognostic factors and management strategies for EPLs, emphasising a multidisciplinary approach to managing EPLs. This paper aims to guide clinicians in managing these challenging cases with a combination of endodontic and periodontal therapies.

## Key points

- Survival rates of endodontic-periodontal lesion-affected teeth are over 85% at five years. As such, attempts should be made to preserve these teeth where possible.
- A staged management approach is often appropriate, where non-surgical root canal treatment and subgingival instrumentation is indicated if there are no significant root fractures, perforations or resorptive lesions.
- Specialist referral is often required due to the need for the use of a dental operating microscope and/or surgical management.

## Introduction

The pulp and the periodontium have an intrinsic relationship from their embryonic origins. Although they frequently present with discrete pathologies, several portals exist between pulp and periodontium that may allow passage of microorganisms between these two compartments (Table 1). Subsequently, disease in one site may contribute to disease in the other, with the potential to coalesce, producing an endodontic-periodontal lesion (EPL).

**Table 1:** Common pathways of communication between the pulp and periodontium

**Anatomical/developmental**	**Acquired/pathological**
Apical foramen of main canal Accessory/lateral canals Dentinal tubules	Cracks Root fractures Root resorptions Iatrogenic damage

The dentino-pulpal complex can directly communicate with the periodontium via several pathways, allowing for bacterial cross-seeding.^1^^,^^2^ Alongside the main root canal, accessory canals are common. Reported prevalence is between 18-79%,^3^^,^^4^ the apical third being the most frequent location, as well as in posterior teeth.^5^ Microscopically, dentinal tubules can become invaded with microorganisms from advancing periodontal lesions, secondary to recession or periodontal therapy, or internally following pulpal necrosis and microbial colonisation of the pulp.

Although these anatomical portals exist, questions have been raised on their relevance in disease spread.

Endodontically, the high prevalence of accessory canals does not match the low frequency of lateral radiolucencies of endodontic origin.^6^ Moreover, studies have demonstrated that even when teeth are non-vital, tissue in accessory canals may not be irreversibly inflamed or contaminated with bacteria. The size and patency of accessory canals may dictate whether inflammation is evoked in adjacent tissues.^7^ Conversely, a series of studies suggested that teeth with apical pathology had a higher correlation with multiple negative periodontal outcome measures.^8^^,^^9^^,^^10^

Diametrically, periodontal disease has not been shown to cause pulpal necrosis until the lesion extends to the apex where the main pulpal blood vessels are compromised. This is usually restricted to the affected root for multirooted teeth.^7^^,^^11^ However, fibrosis, calcification and partial necrosis can be seen within the pulp, correlating to the severity of periodontal disease.^12^^,^^13^ Furthermore, contemporary research has revealed similar molecular inflammatory profiles in the pulps of vital teeth affected by advanced periodontal disease and those with irreversible pulpitis.^14^ Additionally, previously root-filled teeth affected with periodontal disease, where the natural internal defences of the pulp have been lost, show greater risk of endodontic failure.^15^ Together, these findings suggest that the impact of periodontal disease on the pulpal status may be a contributory one to the ‘stressed pulp syndrome',^16^ rather than a sole, definitive cause of pulpal necrosis.

Aberrant anatomy or pathology can also contribute to the development of EPLs. Root damage from fractures, cracks, or iatrogenic damage can create a passageway for bacterial migration between the pulpal and periodontal tissues. Typically, this leads to a local inflammatory response, which may appear clinically as an isolated deep area of attachment loss. Root resorption also often crosses the juncture of the pulp and periodontium.

Apropos of EPLs, local risk factors, such as invaginations of the crown and/or roots^17^ and root grooves,^18^ provide an increased risk for direct communication between the pulp and periodontium, secondary to more sheltered biofilm accumulation and resultant carious lesions or attachment loss. This significantly impacts the prognosis and treatment plan. Further examples of potential risk factors are presented in Box 1.

The remainder of this article will consider the classification, diagnosis and management of EPLs.

Box 1 Examples of local risk factors for periodontal disease progression

- Root grooves
- Invaginations
- Cemental tear
- Accessory root
- Enamel pearls
- Dilaceration
- Fusions/germination

## Classifications

**Fig. 1:**
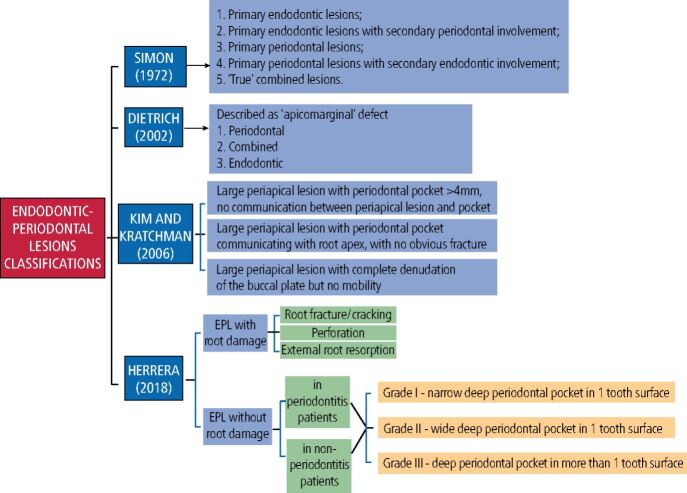
Main EPL classification systems. Image created in BioRender rule below

Several classifications exist for EPLs. The prominent ones are outlined in Fig. 1.^19^^,^^20^^,^^21^^,^^22^ A major criticism of some classifications is that they require determination of the historical course of disease.^19^^,^^21^ A more recent classification developed at the 2017 World Workshop on the Classification of Periodontal and Peri-Implant Diseases and Conditions by Herrera *et al*.^22^ negates the need for determination of the endodontic or periodontal source of the lesion. The authors encourage its use. EPL teeth with ‘root damage' are also considered in this classification. This includes root fractures, iatrogenic perforations, or perforating resorptive lesions.

## Diagnostic pathway

Accurately diagnosing EPL is challenging. The dilemmas frequently faced are determining whether there is pulpal involvement, or for localised cases, whether any root damage is present. 

A thorough assessment is crucial for accurate diagnosis, prognosis and subsequent treatment plan.

### History

The first step in the diagnostic pathway is to take a detailed pain, trauma and dental history. Most EPLs are asymptomatic; however, patients may report pain, swelling, mobility and bad taste. Symptomatic cases may be associated with pulpitis, apical periodontitis, trauma, or iatrogenic damage.^22^

Certain symptoms prompt further evaluation. For instance, pain on release of biting is classically described in cracked teeth. Furthermore, patients with parafunctional habits (bruxism) and heavily restored dentitions are at greater risk of root fractures.

### Clinical examination

Clinical evaluation of EPLs involves assessment of soft and hard tissues.

Soft tissue examination investigates for presence of an abscess/swelling, sinus tract and tenderness on palpation of buccal mucosa, alongside a full-mouth periodontal assessment. A baseline chart reveals the extent of periodontal destruction surrounding the tooth, helping determine the prognosis and monitoring the subsequent success of treatment.

An isolated deep pocket in non-periodontitis patients suggests root damage or disease of endodontic origin without root damage, draining through the gingival sulcus. Furcation involvement in non-periodontitis patients can be secondary to cracks in the pulpal floor or necrotic and infected furcal canals. Therefore, furcal bone loss in a heavily restored tooth may be the first indicator of pulpal necrosis rather than primary periodontal disease.

Hard tissue examination investigates the presence of carious lesions, defective restorations, cracked teeth/root fractures, tenderness on percussion, developmental grooves/anomalies, traumatic occlusal elements and ultimately, tooth restorability.

**Fig. 2:**
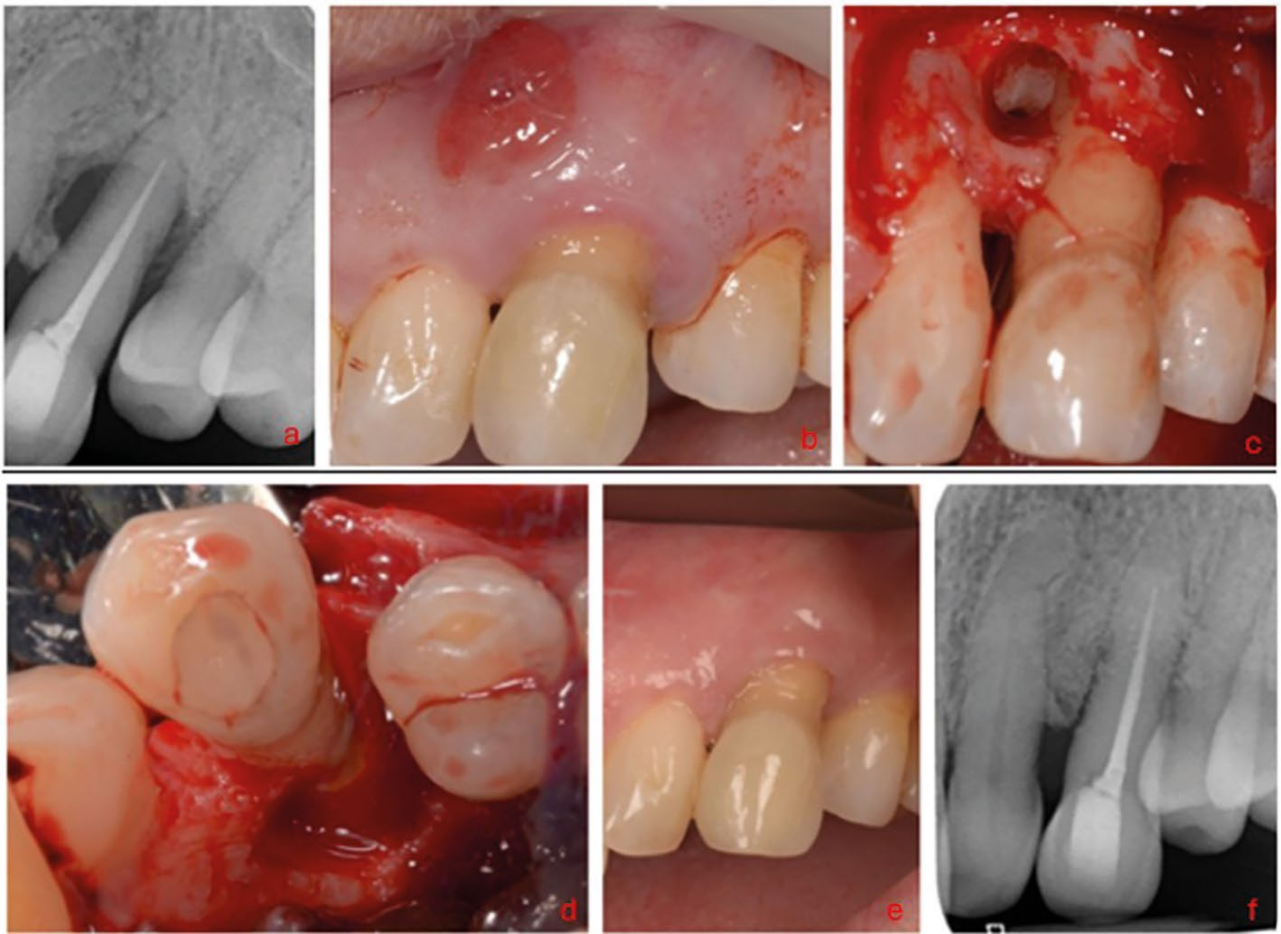
Case of a localised periodontal defect with a buccal sinus affecting tooth 23. a) The PA showed suboptimal obturation of tooth 23, but no frank apical pathology. b) Due to the localised distal probing depth and pattern of bone loss, a root fracture was suspected. Surgical exploration was undertaken and a broad circumferential defect was found circling the mid-third of the root. c, d) The root was stained, but no fracture was visualised. Guided tissue regeneration was undertaken. At one year, the sinus had resolved and the probing depths were ≤4 mm. e, f) Although the initial healing appears positive, the patient was warned of possible future failure given a root fracture was still suspect; although, one was not identified

To aid restorability assessment in the first instance, any restorations and carious lesions should be removed.^23^ Cracks/root fractures can be assessed visually with magnification, transillumination and tooth sleuth testing. Staining with methylene blue dye may aid visual inspection. Pain on release of biting on the tooth sleuth is associated with the presence of a crack. Cracks and root fractures are often hard to detect clinically due to their cleavage planes and may only manifest after accessing the pulp chamber or tooth extraction. If cracks/root fractures are suspected but not confirmed, the patient should be informed that, while treatment can be attempted, the outcome is unpredictable (Fig. 2).

Occlusal assessment of static and dynamic occlusion, premature and heavy contacts, and fremitus should be performed, as pathological occlusal elements can contribute to cracks/root fractures and subsequent EPLs. Where possible, adjustment should be carried out. Pulp sensibility testing using cold and electric pulp testing helps determine tooth innervation but doesn't provide accurate information regarding the blood supply.^24^ Furthermore, multirooted teeth with partial pulp necrosis may give false-positive responses.^25^^,^^26^

### Radiographic examination

This includes evaluation of periapical radiographs (PA) and possibly cone beam computed tomography (CBCT) scans. Where a CBCT scan is required for diagnostic purposes, the authors recommend referral onto an appropriately trained clinician.

**Fig. 3:**
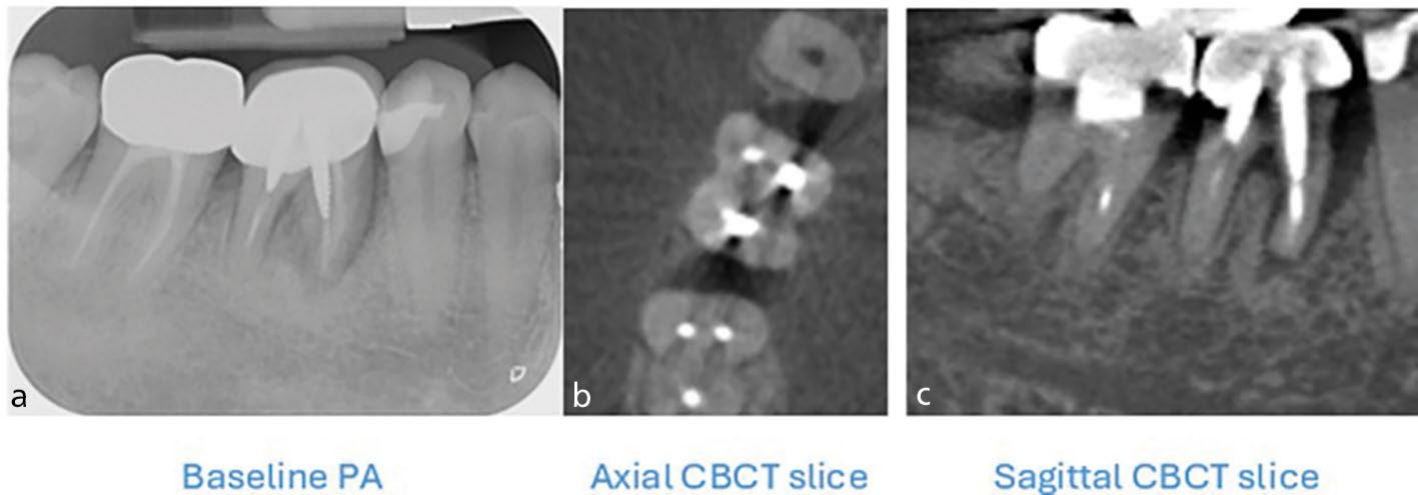
a, b, c) A J-shaped radiolucency, seen in the left image around the mesial root of tooth 46, typically indicates vertical root fracture (VRF). VRF may also present as a halo-shaped radiolucency around the root and involve the furcation of multirooted teeth (right image). Root fractures are often not directly detectable on CBCT imaging due to insufficient voxel size, non-axial cleavage planes and beam hardening artefacts due to restorative materials, such as metal posts (seen as white streaks and black bands in the middle image). It should be noted that a root groove may mimic a VRF radiographically (Fig. 4)

**Fig. 4:**
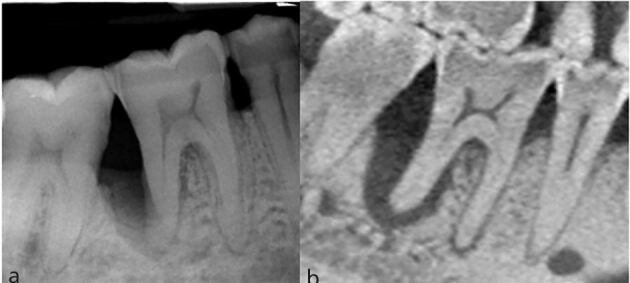
a, b) PA and sagittal slice CBCT showing J-shaped radiolucency associated with the distal root of tooth 46. The distal root in fact had a root groove, rather than a VRF
- Root fractures (to some extent)

PA provides information about the extent of the intrabony defect, quality of root filling and root morphology. Limitations include anatomical noise,^27^ geometric distortion^28^ and a two-dimensional representation of a three-dimensional object.^29^ CBCT provides three-dimensional visualisation of the root anatomy with high geometric accuracy and minimal anatomical noise.^30^ With regards to EPLs, CBCT can help recognise:

- The presence, extent and location of perforations
- Internal/external resorption
- Anatomical variations/anomalies
- The pattern of bone loss around teeth (Fig. 3, Fig. 4)

**Fig. 5:**
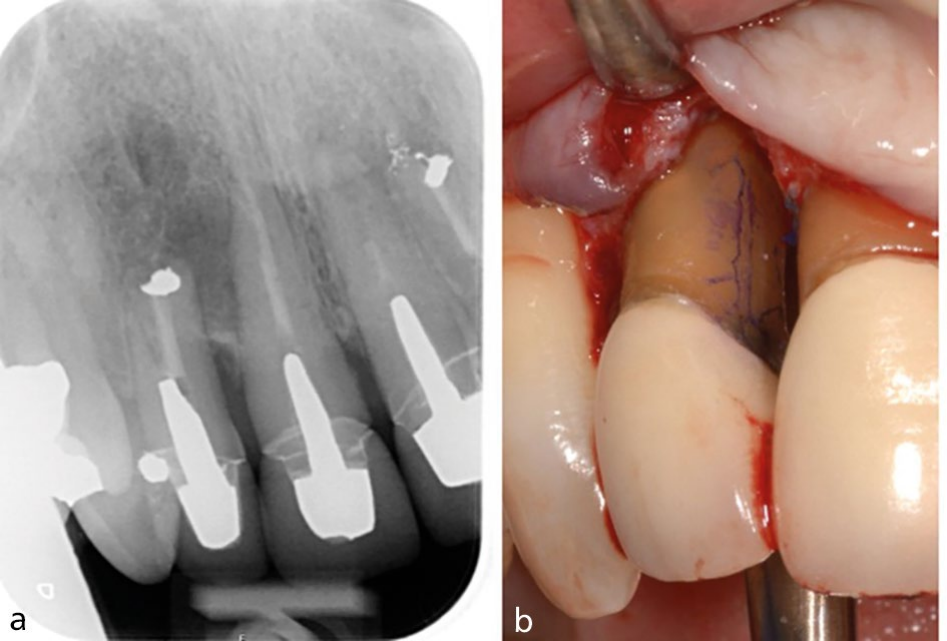
a, b) Pre-op PA of tooth 12 with persistent localised periodontal pocket. Bone loss extends to just below the end of the post. A CBCT was not performed on the basis that substantial scatter would prevent accurate visualisation of any fractures. Surgical exploration was opted for instead, where a root fracture was identified. Additionally, for post-crown restorations, a history of repeated debonding should raise a high suspicion of a root fracture

**Fig. 6:**
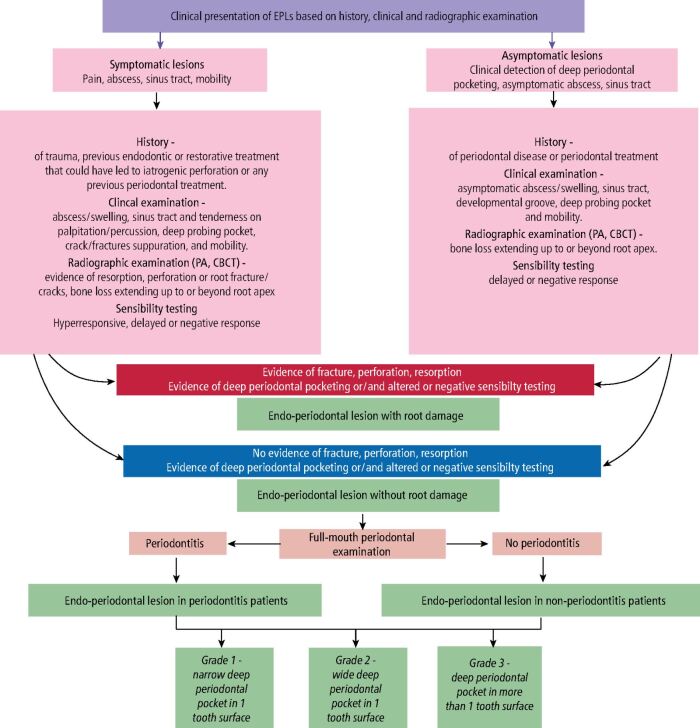
Diagnostic pathway for teeth with endodontic periodontal lesions. Created in BioRender. Moyes, D. (2024) BioRender.com/r60j931

However, beam hardening artefacts due to restorative materials (Figures 3 and 5) are a major drawback of CBCT imaging, reducing the quality and diagnostic value of the image.^31^ CBCT is a valuable tool for assessing EPLs, and facilitating diagnosis, prognosis and clinical decision-making.^32^ However case selection is important and radiation dose reduction protocols should be considered. Once all the clinical information has been gathered, a diagnosis can be made in line with the Herrera *et al*.^22^ classification. Figure 6 summarises the diagnostic pathway.

## Prognosis

‘Survival' rates for EPLs treated surgically are likely between 88.5-92% at five years^33^^,^^34^ and approximately 87% at ten years.^35^ This is significant, as many of the included EPL teeth with bone loss to or beyond the apex would conventionally be considered to have a hopeless-poor prognosis. However, more than half the EPL teeth that survived to five years were splinted long-term to reduce mobility.^34^

Several retrospective and prospective cohort studies using a stricter outcome of ‘success' yielded an approximately 70-80% success rate at up to 12.5 years.^36^^,^^37^^,^^38^^,^^39^^,^^40^

In the cases of both survival and success, the teeth included in the quoted studies were managed surgically. No long-term studies have been conducted on the survival or success of EPL teeth treated non-surgically.

The difference in outcomes between ‘survival' and ‘success' is relevant when discussing various management options with the patient. ‘Survival' is considered the most critical patient-reported outcome measure,^41^ whereas ‘success' is more clinician-centred, with stricter radiographic criteria. The implant literature often quotes ‘success' rates in the range of 90-95% at ten years;^42^^,^^43^ however, the implant criteria for ‘success' is more comparable to tooth ‘survival' rather than tooth ‘success'. As such, the aforequoted ‘survival' rates for EPLs may be comparable long-term to that of implants, with correct case selection; although, the current data is preliminary in this regard. Additionally, when compared to single implants over a ten-year period, maintaining EPL teeth with bone loss past the apex was significantly more cost-effective for patients than having a dental implant.^35^

Prognostic factors for EPL survival, success and improved periodontal outcomes are poorly reported in the literature. While specific evidence related to the prognostic factors of EPLs is sparse, it is also important to consider the ‘specialty specific' prognostic factors when managing these types of cases.^44^^,^^45^ Possible positive prognostic factors are presented in Table 2. These factors are largely derived from studies which managed EPL at least partly surgically, with regenerative or endodontic microsurgery.

**Table 2:** Positive prognostic factors for EPL outcomes

Patient	Younger patients^39^ Female patients^39^ Good plaque control and low full mouth bleeding scores^46^ Non-smokers^47^ Well-controlled diabetes^47^
Oral	No bruxism^48^
Tooth	**Pre-operative factors:** Less baseline attachment loss^49^ Anterior teeth^39^ Maxillary teeth^39^ No or minimal (≤ Grade 1) mobility **Endodontic/restorative intra-operative factors:** Well-sealed cuspal coverage restoration^33^ Orthograde root-filling within 2 mm of the root apex^50^ Bioceramic use as a retro or orthograde root filling material^50^^,^^69^ Undertaking elective RCT on EPL teeth with bone loss to or past the apex, where surgical periodontal treatment is planned^33^^,^^35^^,^^52^ Perforations <3 mm diameter^72^ Single cracks as opposed to multiple cracks in a tooth^54^ Cracked teeth which aren't terminal abutments^54^ Lower volume of any resorptive defect^77^ **Periodontal intra-operative factors:** Avoiding damage to cementum when carrying out periodontal therapy^56^^,^^57^ Use of guided tissue regeneration techniques in two and three-walled defects^34^^,^^48^^,^^58^^,^^69^ Combined endodontic-periodontal intra-operative factors: Treatment with a combination of non-surgical RCT and subgingival PMPR^55^ Subgingival PMPR at the same time as or within three months post-RCT^52^^,^^53^
Defect morphology	Narrow infrabony defect^61^ Contained (three-walled) defects^61^ Decreased extent of vertical component of furcation defects^48^
Operator	Operator skills and experience Minimally invasive surgical technique

## Management strategies

The discussion of management strategies should be prefaced with the disclaimer that the overall quality of evidence for management of EPL is weak. This is because most studies on EPL teeth do not have appropriate control groups, have short follow-up times and mainly include only small cohorts of teeth.

**Fig. 7:**
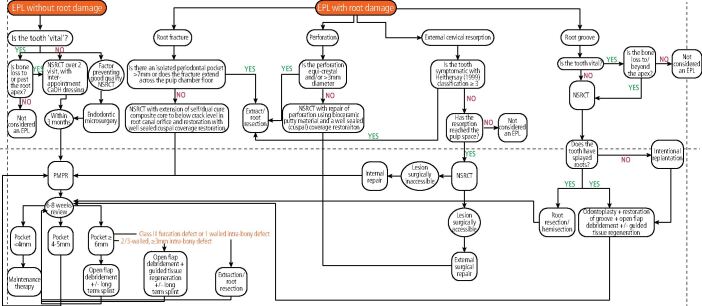
Flowchart illustrating decision-making processes for various management options

**Fig. 8:**
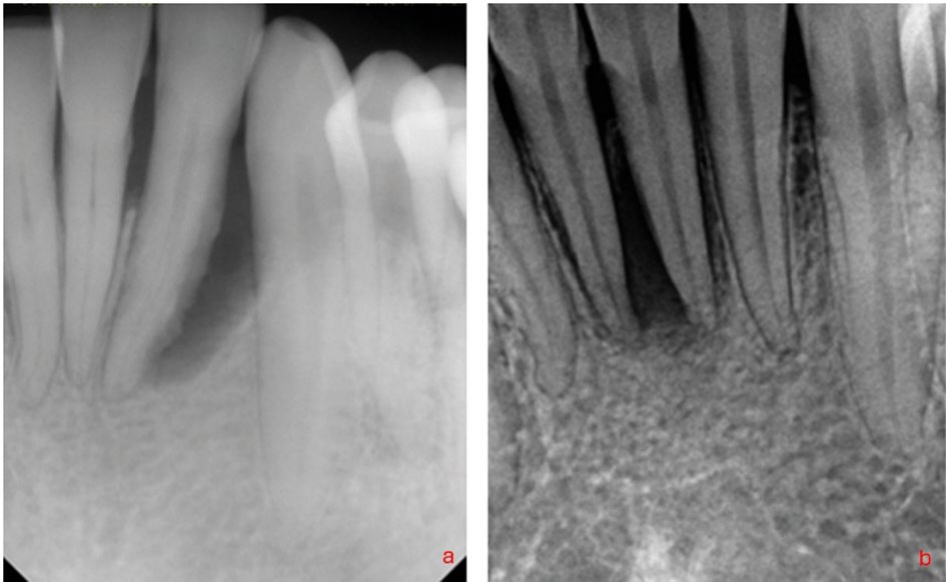
a) Tooth 32 presents with a localised deep periodontal pocket and vertical bone loss extending to, but not encompassing, the apex. A positive response to Endofrost was elicited but suppuration was consistently present. The true status of the pulp is questionable. b) Tooth 31 presented with bone loss to the apex and deep buccal and lingual probing depths. In contrast, both centrals were consistently positive to cold testing and so a decision needs to be made on whether to instigate RCT in this scenario

Management strategies vary according to whether root damage is present and whether the tooth is ‘vital'. There is general agreement that when the pulpal status is confirmed as necrotic, root canal treatment (RCT) should be initiated in the first instance,^51^ unless a significant root fracture, perforation, or resorptive lesion is confirmed (Fig. 7). There is more recent evidence suggesting that in ‘vital' teeth with apicomarginal bone loss to the apex, RCT may be indicated to further improve periodontal probing depths and clinical attachment levels; although, the authors stress that this is currently preliminary evidence.^14^^,^^33^^,^^35^^,^^52^ As such, there will be occasions where the operator has to make a judgement call as to whether RCT should be undertaken when the vitality status is unclear (Fig. 8).

The below sections will discuss the rationale and evidence base for various aspects of management, while Figure 7 illustrates some of the decision-making processes that may be considered when deciding between various management options.

### EPL without root damage

#### Non-surgical management strategies

Non-surgical RCT is indicated in the first instance, according to the results of two systematic reviews.^51^^,^^53^

Additional subgingival professional mechanical plaque removal (PMPR) achieves superior outcomes compared to non-surgical RCT alone.^55^ This should be done using ultrasonics as opposed to hand instruments to minimise the chance of damage to the cementum layer, potentially allowing for greater periodontal ligament re-attachment.^59^^,^^60^

It is unclear what the time gap between subgingival PMPR and non-surgical RCT should be,^62^ with only one study (using surgical periodontal treatment) indicating superior periodontal outcomes if undertaken within three months of non-surgical RCT. As a minimum, subgingival PMPR should be carried out regularly on a long-term basis where the periodontal pocket remains ≥4 mm with bleeding on probing. Once the probing depth is ≤4 mm without bleeding on probing, the patient should be placed on a supportive periodontal care pathway.^63^

**Fig. 9:**
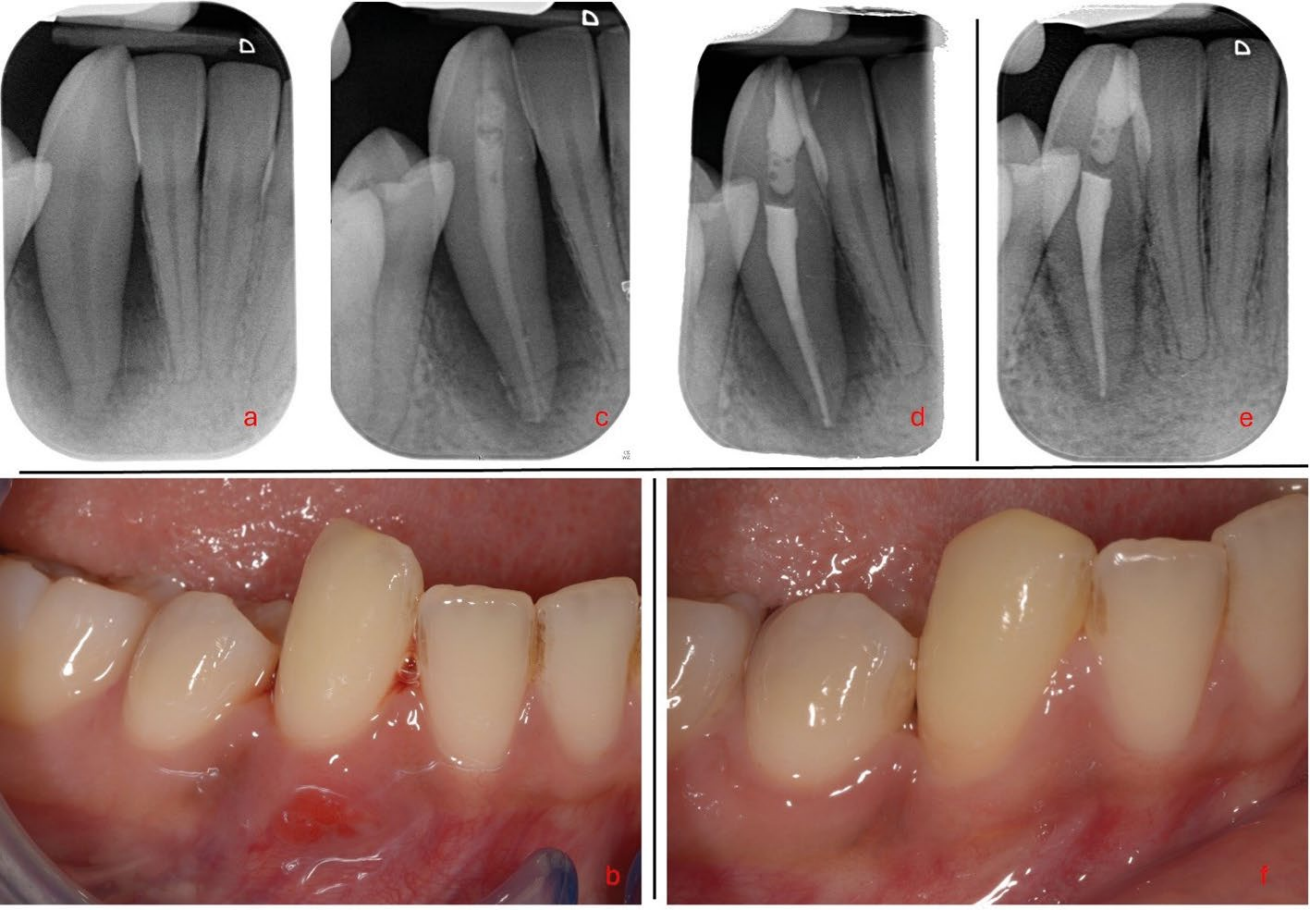
Case of localised periodontitis in a young patient, including an EPL affecting tooth 43. Initial presentation of tooth 43 with deep periodontal probing depths >6 mm affecting buccal lingual and distal aspects of the tooth combined with diffuse apical pathology combined with marginal bone loss. a, b) The tooth was also over-erupted but not mobile. c, d) RCT was undertaken over two visits, with recontouring of the crown to improve appearance. Targeted subgingival PMPR was undertaken immediately after completion of the RCT. e, f) At six-month review, probing depths had reduced to 4 mm with resolution of the inflammation, no bleeding on probing and radiographic bone fill seen. The tooth was placed into a maintenance phase

Non-surgical RCT should be carried out using 0.5-5.25% sodium hypochlorite.^64^ A penultimate rinse with ethylenediaminetetraacetic acid may also be used.^68^ Two-visit endodontic treatment, with an interim dressing of calcium hydroxide, may improve periodontal outcomes^65^^,^^66^ compared to single-visit treatment, with a tentative suggestion that subgingival PMPR should be done while the intra-canal dressing is in situ. The biological basis for this lies in the fact that the initiation of non-surgical RCT and intra-canal dressing reduces the bacterial load and levels of inflammatory mediators, such as lipopolysaccharide, reducing the potential for cross-seeding between necrotic pulp and inflamed periodontal tissues^8^^,^^66^^,^^67^ (Fig. 9).

#### Surgical management strategies

Most studies on EPL employ surgical management strategies.

Surgical management strategies can be considered following the failure of non-surgical management as described above or can be considered immediately as an adjunct to non-surgical RCT. In the authors' opinion, most cases will undergo a non-surgical approach first, followed by review, with only select cases proceeding directly to surgery. Cases that may be more likely to require regenerative surgical approaches include Grade 2 and 3 EPL teeth according to the Herrera *et al*.^22^ classification.^34^^,^^48^^,^^69^^,^^70^^,^^71^ Therefore, referral to an appropriately trained clinician should be considered if this diagnosis is made.

Prerequisites for any surgical approach include:

- No medical contra-indications to surgery
- A motivated patient with satisfactory plaque control and low bleeding scores^63^
- Where non-surgical management is not possible eg long posts.

**Fig. 10:**
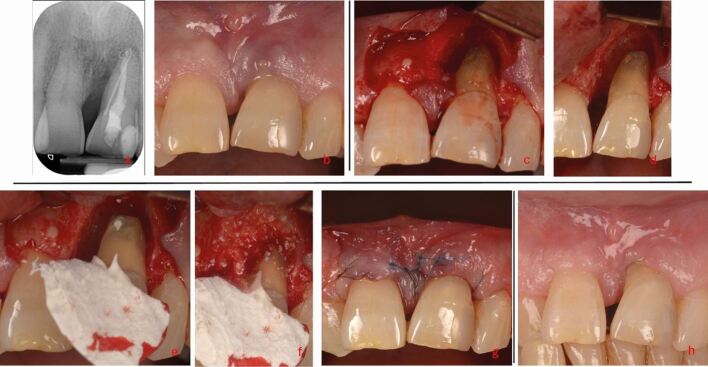
Persistent EPL case following RCT on tooth 21 (apex managed with MTA apexification). a, b) Deep buccal, mesial and palatal probing depths with associated vertical bone loss to the apex were present at baseline. c, d) Following surgical exposure, the granulation tissue was removed and an apicectomy was conducted as the lesion was assumed to be of endodontic origin. d, e) A small perforation was noted as well, which was repaired with a bioceramic putty (Total Fill BC putty). The defect was a contained two-walled defect with a narrow radiographic defect angle <20 ° and so deemed amendable to guided tissue regeneration. f) The defect was filled with a bovine-derived xenograft and collagen membrane (BioOss Collagen and BioGuide). g, h) The immediate post-operative appearance and at one-month review showing resolution of the inflammation with some recession. Due to the increased mobility post-operatively, a splint was placed which was subsequently changed to a lab-made, 0.4 mm, round wire splint, placed palatally for aesthetic reasons

**Fig. 11:**
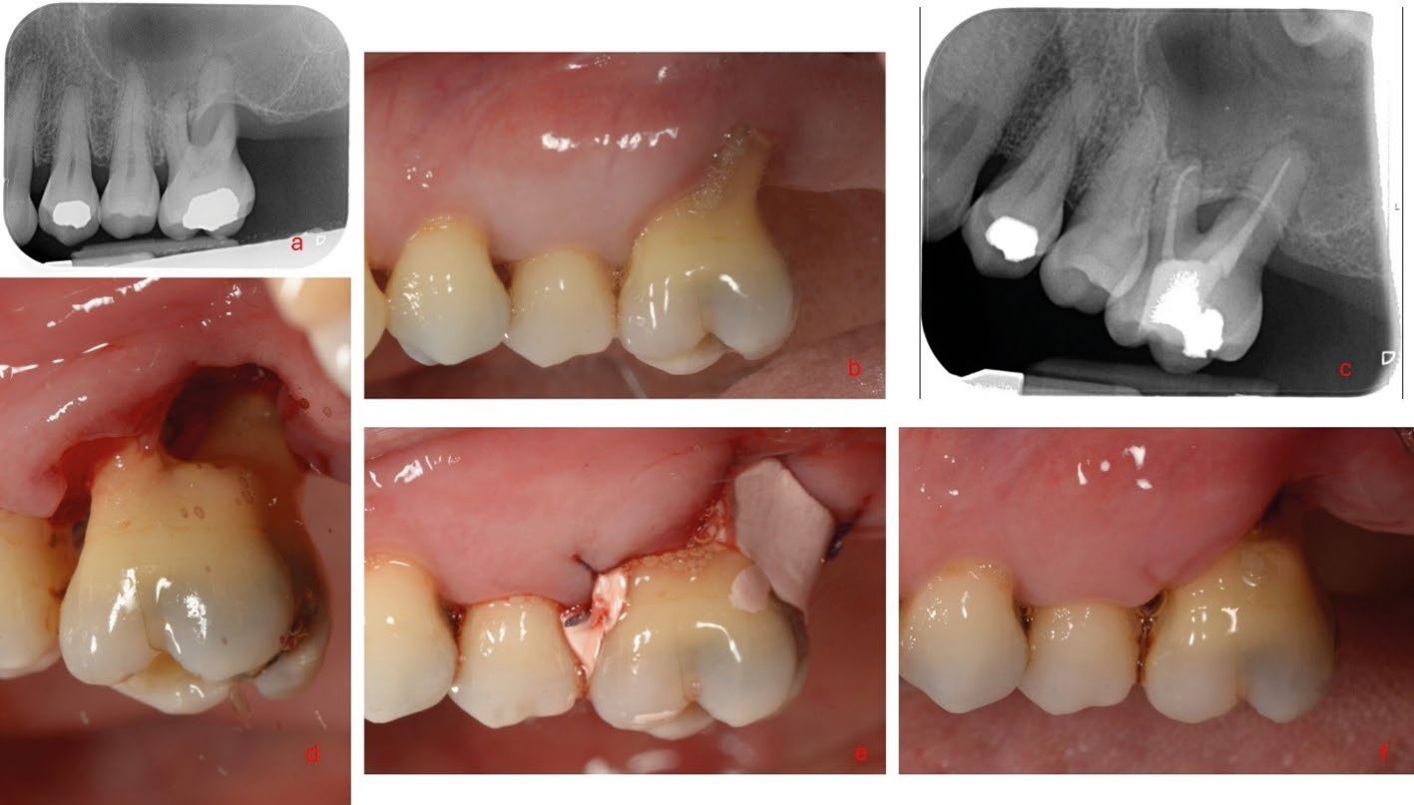
a, b, c) Localised periodontitis case with associated EPL on tooth 26. The distal root shows recession as well as inflammatory root resorption and there is as a degree III furcation lesion. c, d) This case was not amendable to regenerative surgery. In this case, RCT was conducted, followed by resection of the distal root. e) Floss was used to pass a Coepak periodontal dressing through the furcation to try and maintain the space and facilitate regular plaque removal from the furcation area. f) At one month, the furcation is open, but there is still some inflammation present and ongoing supportive care will be needed. This case will likely be classed as survival rather than success

There is no strong evidence on which surgical management strategy is best for EPL teeth as well-controlled studies are not available. Often, the technique employed will depend on the extent of bone loss, defect morphology and root morphology (Fig. 10, Fig. 11). The most employed surgical management strategies include:

- Open flap debridement
- Guided tissue regeneration techniques
- Root resection/hemisection
- Apicectomy

Apicectomy is not considered as part of standard surgical management for EPL unless the endodontic component is not predictably manageable non-surgically.

### EPL with root damage

#### Perforations

Gorni *et al*.^72^ assessed the success rate of perforated EPL teeth which had non-surgical RCT and perforations repaired with mineral trioxide aggregate (MTA) over a 14-year period. At two years, 41/49 (84%) of perforated EPL teeth were ‘successful' following non-surgical RCT and perforation repair, versus 74/75 (99%) of non-EPL teeth, representing a statistically significant difference in success.

By 14 years, the probability of the EPL cohort remaining ‘successful' was only 37%, which again was significantly lower than non-EPL teeth at 72%, with an accelerating failure rate after eight years. This may suggest a lack of stability in MTA as a perforation repair material over the long-term.

It is unclear what the ideal protocol for perforation repair in EPL teeth is. The authors suggest:

- Debridement of the perforation site and the associated periodontal pocket using an ultrasonic scaler. If the perforation is old and has periodontal tissues fungating through it, the tissues may need to be removed using electrocautery
- Disinfection and haemostasis of the perforation site using cotton pellets soaked in sodium hypochlorite 0.5-5.25%
- Repair with a bio-ceramic putty, as opposed to pure MTA, due to their improved mechanical properties.^73^

#### Cracked teeth and fractured roots

Most studies agree that cracked teeth with associated probing defects receiving non-surgical RCT have a reduced survival rate compared to cracked teeth without probing defects.^74^ Interestingly, one study found that cracked teeth with associated periodontal pocket depths of 5-7 mm had statistically similar success and survival rates compared to those with pockets <5 mm. The authors put this down to their restorative protocol, which involved bonding composite into the canal orifices, 2-3 mm below the apical extent of the crack following non-surgical RCT,^75^ followed by placement of a full coverage crown.

A recent systematic review also found that cracked teeth which underwent non-surgical RCT, and didn't have full crowns placed, were 113 times more likely to be extracted than those with full coverage crowns.^76^

Based on these findings, the authors recommend bonding of composite material down the root canal orifice, 2-3 mm below the apical extent of the crack, followed by prompt placement of a full coverage crown in cracked EPL teeth. This may be challenging without the use of a dental operating microscope and heated obturation equipment. As such, referral to an appropriately trained colleague is recommended.

#### Root resorption

Internal or external resorption communicating between the pulp and periodontal tissues ordinarily requires non-surgical RCT with internal or external surgical repair of the resorptive defect using bioceramic materials. The approach for the repair is dictated in most cases by the ease of surgical access to the perforating site and/or the ability to visualise the whole defect completely without a surgical approach.

In cases where resorption is extensive and the patient is symptomatic, extraction is usually indicated. Alternatively, if the patient is asymptomatic, monitoring the lesion would be the management strategy of choice.^77^^,^^78^

Management of EPL teeth with root damage due to resorption is a clinical challenge and should be managed by a clinician with appropriate training and equipment. Referral is usually indicated.

#### Periodontal management

Once the cause of the root damage has been managed, it may be judicious to also remove the biofilm with targeted subgingival PMPR using fine ultrasonic tips and a low-medium power setting to minimise the risk of damage to the cemental layer. In cases where this still fails to stabilise the disease, or surgery has not been employed to access the root damage, then surgical periodontal techniques maybe considered as discussed above.

## Conclusion

The diagnosis and management of EPLs is a clinical challenge. Informed consent is paramount before embarking on the treatment journey, as treatment is often time-consuming, costly and unpredictable. Currently, evidence-based management strategies specific to EPLs are lacking; however, current, long-term survival rates seem promising. Multi-disciplinary management is essential for favourable outcomes and (depending on the aetiology) following a staged approach seems sensible to provide the patient with greater clinical and financial certainty as treatment progresses.

## References

[1] Ahmed H M A, Versiani M A, De-Deus G, Dummer P M H. A new system for classifying root and root canal morphology. *Int Endod J* 2017; **50:** 761-770. 10.1111/iej.12685 27578418

[2] Adriaens P A, Edwards C A, De Boever J A, Loesche W J. Ultrastructural observations on bacterial invasion in cementum and radicular dentin of periodontally diseased human teeth. *J Periodontol* 1988; **59:** 493-503. 10.1902/jop.1988.59.8.493 3171862

[3] Vertucci F J. Root canal anatomy of the human permanent teeth. *Oral Surg Oral Med Oral Pathol* 1984; **58:** 589-599. 10.1016/0030-4220(84)90085-9 6595621

[4] Dammaschke T, Witt M, Ott K, Schäfer E. Scanning electron microscopic investigation of incidence, location, and size of accessory foramina in primary and permanent molars. *Quintessence Int* 2004; **35:** 699-705. 15470993

[5] De Deus Q D. Frequency, location, and direction of the lateral, secondary, and accessory canals. *J Endod* 1975; **1:** 361-366. 10.1016/s0099-2399(75)80211-1 10697487

[6] Meirinhos J, Martins J N, Pereira B, Baruwa A O, Ginjeira A. Prevalence of lateral radiolucency, apical root resorption and periapical lesions in Portuguese patients: a CBCT cross-sectional study with a worldwide overview. *Eur Endod J* 2021; **6:** 56-71. 10.14744/eej.2021.29981 PMC8056814 33762535

[7] Ricucci D, Siqueira J F Jr. Fate of the tissue in lateral canals and apical ramifications in response to pathologic conditions and treatment procedures. *J Endod* 2010; **36:** 1-15. 10.1016/j.joen.2009.09.038 20003929

[8] Ehnevid H, Jansson L, Lindskog S, Blomlöf L. Periodontal healing in teeth with periapical lesions: a clinical retrospective study. *J Clin Periodontol* 1993; **20:** 254-258. 10.1111/j.1600-051x.1993.tb00354.x 8473535

[9] Jansson L, Ehnevid H, Lindskog S, Blomlöf L. Relationship between periapical and periodontal status: a clinical retrospective study. *J Clin Periodontol* 1993; **20:** 117-123. 10.1111/j.1600-051x.1993.tb00325.x 8436630

[10] Jansson L, Ehnevid H, Lindskog S, Blomlöf L. The influence of endodontic infection on progression of marginal bone loss in periodontitis. *J Clin Periodontol* 1995; **22:** 729-734. 10.1111/j.1600-051x.1995.tb00254.x 8682918

[11] Langeland K, Rodrigues H, Dowden W. Periodontal disease, bacteria, and pulpal histopathology. *Oral Surg Oral Med Oral Pathol* 1974; **37:** 257-270. 10.1016/0030-4220(74)90421-6 4520855

[12] Gautam S, Galgali S R, Sheethal H S, Priya N S. Pulpal changes associated with advanced periodontal disease: a histopathological study. *J Oral Maxillofac Pathol* 2017; **21:** 58-63. 10.4103/0973-029X.203795 PMC5406820 28479688

[13] Wan L, Lu H, Xuan D, Yan Y X, Zhang J C. Histological changes within dental pulps in teeth with moderate-to-severe chronic periodontitis. *Int Endod J* 2015; **48:** 95-102. 10.1111/iej.12282 24646359

[14] Louzada L M, Arruda-Vasconcelos R, Kearney M, Yamauchi Y, Gomes B P F A, Duncan H F. Teeth with vital pulps and stage III periodontitis unresponsive to therapy exhibit a pulpal inflammatory profile similar to symptomatic irreversible pulpitis. *Int Endod J* 2024; **57:** 1769-1782. 10.1111/iej.14139 39189896

[15] Ruiz X F, Duran-Sindreu F, Shemesh H *et al*. Development of periapical lesions in endodontically treated teeth with and without periodontal involvement: a retrospective cohort study. *J Endod* 2017; **43:** 1246-1249. 10.1016/j.joen.2017.03.037 28606666

[16] Abou-Rass M. The stressed pulp condition: an endodontic-restorative diagnostic concept. *J Prosthet Dent* 1982; **48:** 264-267. 10.1016/0022-3913(82)90008-7 6750089

[17] Alani A, Bishop K. Dens invaginatus. Part 1: classification, prevalence and aetiology. *Int Endod J* 2008; **41:** 1123-1136. 10.1111/j.1365-2591.2008.01468.x 19133103

[18] Leknes K N, Lie T, Selvig K A. Root grooves: a risk factor in periodontal attachment loss. *J Periodontol* 1994; **65:** 859-863. 10.1902/jop.1994.65.9.859 7990023

[19] Simon J H, Glick D H, Frank A L. The relationship of endodontic-periodontic lesions. *J Periodontol* 1972; **43:** 202-208. 10.1902/jop.1972.43.4.202 4505605

[20] Kim S, Kratchman S. Modern endodontic surgery concepts and practice: a review. *J Endod* 2006; **32:** 601-623. 10.1016/j.joen.2005.12.010 16793466

[21] Dietrich T, Zunker P, Dietrich D, Bernimoulin J-P. Apicomarginal defects in periradicular surgery: classification and diagnostic aspects. *Oral Surg Oral Med Oral Pathol Oral Radiol Endodontol* 2002; **94:** 233-239. 10.1067/moe.2002.123864 12221392

[22] Herrera D, Retamal-Valdes B, Alonso B, Feres M. Acute periodontal lesions (periodontal abscesses and necrotizing periodontal diseases) and endo-periodontal lesions. *J Clin Periodontol* 2018; DOI: 10.1002/JPER.16-0642. 10.1111/jcpe.12941 29926493

[23] Abbott P V. Assessing restored teeth with pulp and periapical diseases for the presence of cracks, caries and marginal breakdown. *Aust Dent J* 2004;**49:** 33-39. 10.1111/j.1834-7819.2004.tb00047.x 15104132

[24] Mainkar A, Kim S G. Diagnostic accuracy of 5 dental pulp tests: a systematic review and meta-analysis. *J Endod* 2018; **44:** 694-702. 10.1016/j.joen.2018.01.021 29571914

[25] Peters D D, Baumgartner J C, Lorton L. Adult pulpal diagnosis. I. Evaluation of the positive and negative responses to cold and electrical pulp tests. *J Endod* 1994; **20:** 506-511. 10.1016/S0099-2399(06)80048-8 7714424

[26] Petersson K, Söderström C, Kiani-Anaraki M, Lévy G. Evaluation of the ability of thermal and electrical tests to register pulp vitality. *Endod Dent Traumatol* 1999; **15:** 127-131. 10.1111/j.1600-9657.1999.tb00769.x 10530156

[27] Cotton T P, Geisler T M, Holden D T, Schwartz S A, Schindler W G. Endodontic applications of cone-beam volumetric tomography. *J Endod* 2007; **33:** 1121-1132. 10.1016/j.joen.2007.06.011 17931947

[28] Forsberg J, Halse A. Radiographic simulation of a periapieal lesion comparing the paralleling and the bisecting-angle techniques. *Int Endod J* 1994; **27:** 133-138. 10.1111/j.1365-2591.1994.tb00242.x 7995645

[29] Webber R L, Messura J K. An *in vivo* comparison of diagnostic information obtained from tuned-aperture computed tomography and conventional dental radiographic imaging modalities. *Oral Surg Oral Med Oral Pathol Oral Radiol Endodontol* 1999; **88:** 239-247. 10.1016/s1079-2104(99)70122-8 10468470

[30] Patel S. New dimensions in endodontic imaging: part 2. Cone beam computed tomography. *Int Endod J* 2009; **42:** 463-475. 10.1111/j.1365-2591.2008.01531.x 19298576

[31] Lofthag-Hansen S, Huumonen S, Gröndahl K, Gröndahl H-G. Limited cone-beam CT and intraoral radiography for the diagnosis of periapical pathology. *Oral Surg Oral Med Oral Pathol Oral Radiol Endodontol* 2007; **103:** 114-119. 10.1016/j.tripleo.2006.01.001 17178504

[32] Patil B A, Shakeel S. Evaluation of endo perio lesions using cone beam computed tomography (CBCT) - a cross sectional study. *Int J Periodontics Restorative Dent* 2023; DOI: 10.11607/prd.6380. 10.11607/prd.6380 37552178

[33] Oh S, Chung S H, Han J-Y. Periodontal regenerative therapy in endo-periodontal lesions: a retrospective study over 5 years. *J Periodontal Implant Sci* 2019; **49:** 90-104. 10.5051/jpis.2019.49.2.90 PMC6494774 31098330

[34] Cortellini P, Stalpers G, Mollo A, Tonetti M S. Periodontal regeneration versus extraction and prosthetic replacement of teeth severely compromised by attachment loss to the apex: 5-year results of an ongoing randomized clinical trial. *J Clin Periodontol* 2011; **38:** 915-924. 10.1111/j.1600-051X.2011.01768.x 21777268

[35] Cortellini P, Stalpers G, Mollo A, Tonetti M S. Periodontal regeneration versus extraction and dental implant or prosthetic replacement of teeth severely compromised by attachment loss to the apex: a randomized controlled clinical trial reporting 10-year outcomes, survival analysis and mean cumulative cost of recurrence. *J Clin Periodontol* 2020; **47:** 768-776. 10.1111/jcpe.13289 PMC7384072 32249446

[36] Kim E, Song J-S, Jung I-Y, Lee S-J, Kim S. Prospective clinical study evaluating endodontic microsurgery outcomes for cases with lesions of endodontic origin compared with cases with lesions of combined periodontal-endodontic origin. *J Endod* 2008; **34:** 546-551. 10.1016/j.joen.2008.01.023 18436032

[37] Song M, Kim H-C, Lee W, Kim E. Analysis of the cause of failure in nonsurgical endodontic treatment by microscopic inspection during endodontic microsurgery. *J Endod* 2011; **37:** 1516-1519. 10.1016/j.joen.2011.06.032 22000454

[38] Song M, Chung W, Lee S-J, Kim E. Long-term outcome of the cases classified as successes based on short-term follow-up in endodontic microsurgery. *J Endod* 2012; **38:** 1192-1196. 10.1016/j.joen.2012.06.014 22892734

[39] Song M, Kim S G, Lee S-J, Kim B, Kim E. Prognostic factors of clinical outcomes in endodontic microsurgery: a prospective study. *J Endod* 2013; **39:** 1491-1497. 10.1016/j.joen.2013.08.026 24238435

[40] Song M, Kang M, Kang D R, Jung H I, Kim E. Comparison of the effect of endodontic-periodontal combined lesion on the outcome of endodontic microsurgery with that of isolated endodontic lesion: survival analysis using propensity score analysis. *Clin Oral Investig* 2018; **22:** 1717-1724. 10.1007/s00784-017-2265-1 29098442

[41] Duncan H F, Nagendrababu V, El-Karim I A, Dummer P M H. Outcome measures to assess the effectiveness of endodontic treatment for pulpitis and apical periodontitis for use in the development of European Society of Endodontology S3 level clinical practice guidelines: a consensus-based development. *Int Endod J* 2021; **54:** 2184-2194. 10.1111/iej.13627 34553383

[42] Howe M-S, Keys W, Richards D. Long-term (10-year) dental implant survival: A systematic review and sensitivity meta-analysis. *J Dent* 2019; **84:** 9-21. 10.1016/j.jdent.2019.03.008 30904559

[43] Moraschini V, Poubel L A, Ferreira V F, Barboza Edos E. Evaluation of survival and success rates of dental implants reported in longitudinal studies with a follow-up period of at least 10 years: a systematic review. *Int J Oral Maxillofac Surg* 2015; **44:** 377-388. 10.1016/j.ijom.2014.10.023 25467739

[44] Cortellini P, Tonetti M S. Clinical concepts for regenerative therapy in intrabony defects. *Periodontol 2000* 2015; **68:** 282-307. 10.1111/prd.12048 25867990

[45] Gulabivala K, Ng Y L. Factors that affect the outcomes of root canal treatment and retreatment - a reframing of the principles. *Int Endod J* 2023; **56:** 82-115. 10.1111/iej.13897 36710532

[46] Chapple I L, Van der Weijden F, Doerfer C *et al*. Primary prevention of periodontitis: managing gingivitis. *J Clin Periodontol* 2015; DOI: 10.1111/jcpe.12366. 10.1111/jcpe.12366 25639826

[47] Ramseier C A, Woelber J P, Kitzmann J, Detzen L, Carra M C, Bouchard P. Impact of risk factor control interventions for smoking cessation and promotion of healthy lifestyles in patients with periodontitis: a systematic review. *J Clin Periodontol* 2020; **47:** 90-106. 10.1111/jcpe.13240 31912512

[48] Tietmann C, Tezer I, Youssef E, Jepsen S, Jepsen K. Management of teeth with Grade 3 endo-periodontal lesions by combined endodontic and regenerative periodontal therapy. *J Clin Med* 2023; **13:** 93. 10.3390/jcm13010093 PMC10779476 38202100

[49] Sarnadas M, Marques J A, Baptista I P, Santos J M. Impact of periodontal attachment loss on the outcome of endodontic microsurgery: a systematic review and meta-analysis. *Medicina (Kaunas)* 2021; **57:** 922. 10.3390/medicina57090922 PMC8465214 34577845

[50] Song M, Jung I-Y, Lee S-J, Lee C-Y, Kim E. Prognostic factors for clinical outcomes in endodontic microsurgery: a retrospective study. *J Endod* 2011; **37:** 927-933. 10.1016/j.joen.2011.04.005 21689546

[51] Schmidt J C, Walter C, Amato M, Weiger R. Treatment of periodontal-endodontic lesions-a systematic review. *J Clin Periodontol* 2014; **41:** 779-790. 10.1111/jcpe.12265 24766568

[52] Dong T, Zhang Y, Li X. Time-lapse between periodontal regeneration surgery and root canal therapy in sever combined periodontal-endodontic lesions. *Saudi Dent J* 2023; **35:** 191-196. 10.1016/j.sdentj.2022.12.009 PMC10024095 36942208

[53] Friedrich F, Scalabrin S A, Weissheimer T *et al*. Influence of the timing of periodontal intervention on periapical/periodontal repair in endodontic-periodontal lesions: a systematic review. *Clin Oral Investig* 2023; **27:** 933-942. 10.1007/s00784-022-04849-4 36585525

[54] Leong D J X, de Souza N N, Sultana R, Yap A U. Outcomes of endodontically treated cracked teeth: a systematic review and meta-analysis. *Clin Oral Investig* 2020; **24:** 465-473. 10.1007/s00784-019-03139-w 31797172

[55] Yan H, Mao X, Hu F, Liu J, Wang J. Observation on the effect of periodontal treatment on patients with combined periodontal-pulpal lesions. *Am J Transl Res* 2021; **13:** 11938-11942. PMC8581903 34786125

[56] Blomlöf L, Lindskog S, Hammarström L. Influence of pulpal treatments on cell and tissue reactions in the marginal periodontium. *J Periodontol* 1988; **59:** 577-583. 10.1902/jop.1988.59.9.577 3183919

[57] Suvan J, Leira Y, Moreno Sancho F M, Graziani F, Derks J, Tomasi C. Subgingival instrumentation for treatment of periodontitis: a systematic review. *J Clin Periodontol* 2020; **47:** 155-175. 10.1111/jcpe.13245 31889320

[58] Nibali L, Koidou V P, Nieri M, Barbato L, Pagliaro U, Cairo F. Regenerative surgery versus access flap for the treatment of intra-bony periodontal defects: a systematic review and meta-analysis. *J Clin Periodontol* 2020; **47:** 320-351. 10.1111/jcpe.13237 31860134

[59] Kawashima H, Sato S, Kishida M, Ito K. A comparison of root surface instrumentation using two piezoelectric ultrasonic scalers and a hand scaler *in vivo*. *J Periodontal Res* 2007; **42:** 90-95. 10.1111/j.1600-0765.2006.00924.x 17214645

[60] Bozbay E, Dominici F, Gokbuget A Y *et al*. Preservation of root cementum: a comparative evaluation of power-driven versus hand instruments. *Int J Dent Hyg* 2018; **16:** 202-209. 10.1111/idh.12249 27860247

[61] Nibali L, Sultan D, Arena C, Pelekos G, Lin G-H, Tonetti M. Periodontal infrabony defects: systematic review of healing by defect morphology following regenerative surgery. *J Clin Periodontol* 2021; **48:** 101-114. 10.1111/jcpe.13381 33025619

[62] Gupta S, Tewari S, Tewari S, Mittal S. Effect of time lapse between endodontic and periodontal therapies on the healing of concurrent endodontic-periodontal lesions without communication: a prospective randomized clinical trial. *J Endod* 2015; **41:** 785-790. 10.1016/j.joen.2015.02.015 25817213

[63] West N, Chapple I, Claydon N *et al*. BSP implementation of European S3-level evidence-based treatment guidelines for stage I-III periodontitis in UK clinical practice. *J Dent* 2021; **106:** 103562. 10.1016/j.jdent.2020.103562 33573801

[64] Ruksakiet K, Hanák L, Farkas N *et al*. Antimicrobial efficacy of chlorhexidine and sodium hypochlorite in root canal disinfection: a systematic review and meta-analysis of randomized controlled trials. *J Endod* 2020; **46:** 1032-1041. 10.1016/j.joen.2020.05.002 32413440

[65] Raheja J, Tewari S, Tewari S, Duhan J. Evaluation of efficacy of chlorhexidine intracanal medicament on the periodontal healing of concomitant endodontic-periodontal lesions without communication: an interventional study. *J Periodontol* 2014; **85:** 1019-1026. 10.1902/jop.2014.130430 24835418

[66] Duque T M, Prado M, Herrera D R, Gomes B P F A. Periodontal and endodontic infectious/inflammatory profile in primary periodontal lesions with secondary endodontic involvement after a calcium hydroxide-based intracanal medication. *Clin Oral Investig* 2019; **23:** 53-63. 10.1007/s00784-018-2401-6 29572688

[67] Ehnevid H, Jansson L E, Lindskog S F, Blomlöf L B. Periodontal healing in relation to radiographic attachment and endodontic infection. *J Periodontol* 1993; **64:** 1199-1204. 10.1902/jop.1993.64.12.1199 8106946

[68] Rossi-Fedele G, Rödig T. Effectiveness of root canal irrigation and dressing for the treatment of apical periodontitis: a systematic review and meta-analysis of clinical trials. *Int Endod J* 2023; **56:** 422-435. 10.1111/iej.13777 35579074

[69] AlJasser R, Bukhary S, AlSarhan M, Alotaibi D, AlOraini S, Habib S R. Regenerative therapy modality for treatment of true combined endodontic-periodontal lesions: a randomized controlled clinical trial. *Int J Environ Res Public Health* 2021; **18:** 6220. 10.3390/ijerph18126220 PMC8227857 34201328

[70] Rohilla R, Tewari S, Nayyar A S. Efficacy of guided tissue regeneration (GTR) membranes in the healing of apico-marginal defects: a prospective, controlled clinical trial. *Int J Orofac Res* 2017; **2:** 11-17.

[71] Ustaoğlu G, Aydin Z U, Özelçi F. Comparison of GTR, T-PRF and open-flap debridement in the treatment of intrabony defects with endo-perio lesions: a randomized controlled trial. *Med Oral Patol Oral Cir Bucal* 2020; DOI: 10.4317/medoral.23231. 10.4317/medoral.23231 PMC6982990 31880284

[72] Gorni F G, Ionescu A C, Ambrogi F, Brambilla E, Gagliani M M. Prognostic factors and primary healing on root perforation repaired with MTA. a 14-year longitudinal study. *J Endod* 2022; **48:** 1092-1099. 10.1016/j.joen.2022.06.005 35714727

[73] Al-Nazhan S, El Mansy I, Al-Nazhan N, Al-Rowais N, Al-Awad G. Outcomes of furcal perforation management using mineral trioxide aggregate and biodentine: a systematic review. *J Appl Oral Sci* 2022; **30:** e20220330. 10.1590/1678-7757-2022-0330 PMC9724492 36477558

[74] Olivieri C V, Raybaud H, Tonoyan L *et al*. Epstein-Barr virus-infected plasma cells in periodontitis lesions. *Microb Pathog* 2020; **143:** 104128. 10.1016/j.micpath.2020.104128 32165332

[75] Davis M C, Shariff S S. Success and survival of endodontically treated cracked teeth with radicular extensions: a 2-to 4-year prospective cohort. *J Endod* 2019; **45:** 848-855. 10.1016/j.joen.2019.03.015 31122690

[76] Zhang S, Xu Y, Ma Y, Zhao W, Jin X, Fu B. The treatment outcomes of cracked teeth: a systematic review and meta-analysis. *J Dent* 2024; **142:** 104843. 10.1016/j.jdent.2024.104843 38272437

[77] Mavridou A M, Rubbers E, Schryvers A *et al*. A clinical approach strategy for the diagnosis, treatment and evaluation of external cervical resorption. *Int Endod J* 2022; **55:** 347-373. 10.1111/iej.13680 35034370

[78] Jebril A, Aljamani S, Jarad F. The surgical management of external cervical resorption: a retrospective observational study of treatment outcomes and classifications. *J Endod* 2020; **46:** 778-785. 10.1016/j.joen.2020.03.006 32334857

